# Assessment of Quality of Life and Stomatognathic Dysfunction in Patients with Maxillofacial Defects Before Orthognathic Surgery

**DOI:** 10.3390/life15050770

**Published:** 2025-05-12

**Authors:** Magdalena Gębska, Zuzanna Sobczyk, Łukasz Pałka, Dorota Margula-Jaśkowska, Konrad Olszewski, Łukasz Kołodziej, Katarzyna Weber-Nowakowska, Robert Kowalczyk, Piotr Seweryn, Bartosz Dalewski

**Affiliations:** 1 Department of Rehabilitation Musculoskeletal System, Pomeranian Medical University, 70-204 Szczecin, Polandkatarzyna.weber.nowakowska@pum.edu.pl (K.W.-N.); 2 Students’ Scientific Association of the Department of Rehabilitation Musculoskeletal System, Pomeranian Medical University, 70-204 Szczecin, Poland; 3 Private Dental Practice, 68-200 Zary, Poland; 4 Maxillo-Facial Surgery Clinic, Pomeranian Medical University, 71-252 Szczecin, Poland; 5 Department of Experimental Dentistry, Faculty of Dentistry, Wroclaw Medical University, 50-425 Wroclaw, Poland; piotr.seweryn@student.umw.edu.pl; 6 Department of Dental Prosthetics, Pomeranian Medical University, 70-204 Szczecin, Poland

**Keywords:** quality of life, maxillofacial deformities, stomatognathic system, orthognathic surgery

## Abstract

Background: Individuals with maxillofacial deformities are concerned not only with their facial appearance but also experience dysfunctions of the stomatognathic system, including mastication, swallowing, speech, and breathing. These impairments may lead to negative psychological responses and a reduced quality of life. Aim: The aim of this study was to assess the quality of life and analyse reported dysfunctions of the stomatognathic system in orthognathic patients prior to surgical intervention. Material and methods: The study group (SG) comprised 63 patients with maxillofacial deformities scheduled for orthognathic surgery. The control group (CG) consisted of 70 patients with malocclusions undergoing orthodontic treatment who did not meet the criteria for surgical intervention. Quality of life was assessed in all participants using the Orthognathic Quality of Life Questionnaire (OQLQ), along with a self-reported questionnaire evaluating the presence of stomatognathic system dysfunctions (SS). Results: Significant differences were observed between the study groups regarding all quality-of-life indicators for orthodontic and orthognathic patients. Patients in the SG more frequently reported difficulties in the specified stomatognathic system functions compared to those in the CG. No statistically significant differences were found between SG patients with Class II and Class III malocclusions in terms of OQLQ scores or the frequency of reported SS dysfunctions. Regarding sex differences, women reported a lower overall quality of life and scored lower in social dimensions related to facial deformity and aesthetics compared to men. No significant correlations were observed between the age of orthognathic patients and quality-of-life assessment. Conclusions 1. Orthognathic patients exhibit a poorer quality of life and a higher prevalence of stomatognathic system dysfunctions compared to patients with malocclusions who do not require surgery. The type of skeletal deformity in surgical patients does not influence quality of life or the frequency of stomatognathic dysfunctions. 2. Unlike age, the patient’s sex is a significant factor in the quality of life before orthognathic surgery.

## 1. Introduction 

In daily clinical practice, in addition to assessing the patient’s biological condition, attention is also given to their emotional state, well-being, and daily functioning. The interrelationships between these aspects and somatic complaints make quality-of-life assessment an essential component of therapeutic management. This is particularly relevant for patients with craniofacial deformities [1,2,3].

Oral Health-Related Quality of Life (OHQoL) defines the extent to which the health status of the oral cavity and perioral tissues affects an individual’s daily functioning [4,5,6]. A healthy oral cavity enables proper food intake, breathing, speech, and social interactions without pain, discomfort, or embarrassment. Good oral health contributes to a patient’s overall well-being [7]. Class II and III malocclusions can significantly impair quality of life, both functionally and aesthetically [8,9].

Maxillofacial deformities fall within the field of craniofacial surgery. Their treatment often requires orthognathic surgery to restore proper occlusal function and achieve facial harmony [10,11]. The preparation process for surgery takes approximately two years and requires close collaboration between specialists, including orthodontists and maxillofacial surgeons [12]. The global prevalence of malocclusions is 56%, with no significant sex-related differences. The highest prevalence rates are observed in Africa (81%) and Europe (72%), followed by the Americas (53%) and Asia (48%) [13]. Precise data on the number of orthognathic surgeries performed worldwide remain limited. It is estimated that approximately 2% of the U.S. population has a malocclusion or facial deformity that could be surgically corrected [14]. In Denmark, 1000 such procedures were performed in 2015, accounting for approximately 0.02% of the country’s population [15,16].

**Figure 1:**
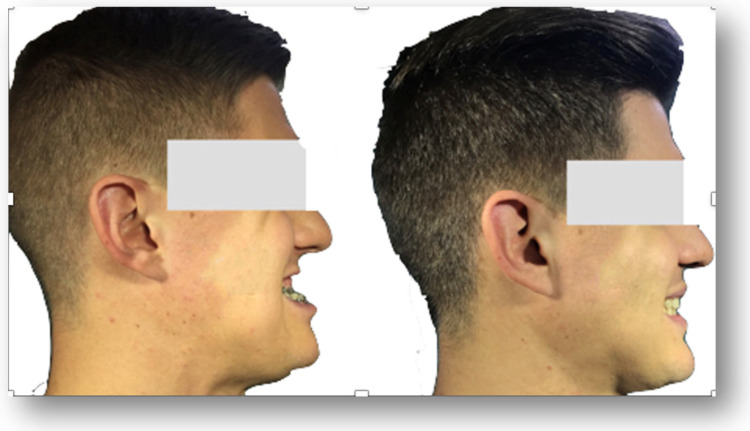
Patient with skeletal class III before and after orthognathic surgery (own source).

Craniofacial deformities can cause both functional and aesthetic complications. Functional issues include difficulties with chewing, biting, swallowing, and speech, as well as deteriorating dental health. In addition, craniofacial deformities may compromise airway patency, contributing to chronic mouth breathing and obstructive sleep apnea, especially in cases involving midfacial or mandibular deficiencies or maxillary arch narrowing [17]. Patients may also experience temporomandibular joint (TMJ) disorders, such as pain, limited joint mobility, and acoustic symptoms [18]. Aesthetic concerns frequently lead to significant social and psychological difficulties [19]. The most commonly reported aesthetic concerns include an excessively long or short face, facial asymmetry, mandibular elongation or shortening, altered chin prominence, excessive gingival exposure, and facial expression disturbances (Figure 1) [20].

Orthognathic surgery is performed to restore the anatomical relationships between the maxillary and mandibular bones, improving occlusal function and preventing further dental damage [21,22]. It can significantly enhance patients’ quality of life, boost self-esteem and self-confidence, and contribute to overall well-being [23].

The indication for combined orthodontic surgical treatment is a skeletal maxillofacial defect. The maxillofacial surgeon determines the extent of the operation by taking into account the physiognomic features (occlusal conditions) and the potential for aesthetic change during the operation. It is usually an unfavourable appearance that motivates someone to seek treatment. It is estimated that 80% of patients seek professional help for aesthetic reasons rather than for health or improved function [24]. Therefore, in this study, we sought to answer the following question: what is the level of quality of life and frequency of stomatognathic disorders in patients being prepared for orthognathic surgery?

### 1.1. Research Questions

1. Do orthognathic patients differ from orthodontically treated patients without surgical intervention in terms of quality-of-life assessment?
2. Do orthognathic patients differ from orthodontically treated patients without surgical intervention in terms of the frequency of specific stomatognathic system dysfunctions?
3. Do orthognathic patients differ from orthodontic patients in the number of reported stomatognathic system dysfunctions?
4. Do orthognathic patients with retrognathia (Class II) differ from those with prognathism (Class III) in terms of quality-of-life assessment?
5. Do orthognathic patients with prognathism differ from those with retrognathia in terms of the frequency of specific stomatognathic system dysfunctions?
6. Are sex and age associated with quality-of-life assessment among orthognathic patients?

The aim of the study was to assess the quality of life and analyse reported dysfunctions of the stomatognathic system (SS) in orthognathic patients prior to surgical intervention.

Specific aims:

1. To compare the quality of life in the areas of facial aesthetics, oral function, perception of dentofacial deformity and social aspects in a group of orthognathic patients and orthodontic patients without indications for surgery.
2. To determine the prevalence and incidence of individual disorders of stomatognathic function in a group of orthognathic and orthodontic patients without indications for surgery.
3. To demonstrate the influence of the type of skeletal class of occlusion (Class II and Class III) on the quality of life in the areas of facial aesthetics, oral function, the perception of dental–facial deformity and in the social aspect, as well as the frequency of occurrence of individual dysfunctions of the stomatognathic system in orthognathic patients.
4. To estimate the influence of socio-demographic variables (gender and age) on the quality of life in a group of orthognathic patients.

### 1.2. Hypothesis

Patients with malocclusions who qualify for surgical intervention, due to the complexity of their health condition, exhibit a lower quality of life than those undergoing conventional orthodontic treatment. Additionally, they present a higher prevalence of stomatognathic system (SS) dysfunctions.

## 2. Material and Methods

The study group (SG) consisted of 63 randomly selected patients (46 women and 27 men) with Class II malocclusion (retrognathia, n = 34) and Class III malocclusion (prognathism, n = 39), who provided informed consent to participate in the study before undergoing surgical treatment. The mean age in the SG was 28.56 years. All respondents were hospitalized at the Department of Maxillofacial Surgery, University Clinical Hospital No. 1 in Szczecin, Poland. Each patient was examined on the day of admission to the department, one day before the planned orthognathic surgery. Inclusion criteria for the study were as follows: age between 18 and 40 years, qualified for orthognathic treatment (skeletal class II or III), qualified for single- or double-jaw surgery, and consent to participate in the study. Patients who were scheduled for either SARPE or genioplasty as the only surgical procedure were excluded. Patients operated on for syndactyly defects, patients operated on for soft and/or hard tissue clefts of the facial cranium and patients undergoing orthognathic treatment after trauma were also excluded. Patients with a history of mental illness and patients undergoing reoperation in the stomatognathic system were also excluded.

The control group (CG) comprised 70 patients (36 women and 24 men) with malocclusions undergoing orthodontic treatment without indications for surgical intervention. The mean age in the CG was 25.28 years. Inclusion criteria for the control group included the following: patients undergoing orthodontic treatment, no indication for orthognathic surgery, no history of orthodontic treatment, and consent to participate in the study. Patients under 18 years of age and those with known mental illness, autoimmune disease, pregnant women, and those taking pharmacological treatment, i.e., painkillers, myorelaxants, etc., were excluded.

The required sample size was 128 people, assuming moderate power (0.50 < Cohen’s d < 0.80), a first-order probability of error α = 5%, and 80% power (a 20% second-order probability of error); calculations were performed using G*Power 3.1 software.

All participants who consented to participate in the study underwent an assessment of quality of life and the presence of stomatognathic system (SS) dysfunctions. The following research tools were used for the assessment:

1. Cunningham et al. developed a special evaluation survey to measure the QoL of patients with severe skeletal deformities [25]. This questionnaire is the Orthognathic Quality of Life Questionnaire (OQLQ). The original form consists of 22 items. This survey aimed to evaluate the effect of dentofacial deformity on the patients’ quality of life in 4 main areas. These areas are facial aesthetics (items 1, 7, 10, 11, and 14, ranging from 0 to 20), oral function (2 to 6, ranging from 0 to 20), dentofacial aesthetic awareness (8, 9, 12, and 13, ranging from 0 to16), and social aspects of dentofacial deformity (items 15 to 22, ranging from 0 to 32). These items are also measured on a 5-point Likert scale, including the following: 0 points: this sentence does not apply to you, or you are not bothered by the dentofacial deformity; 1 point: you are a little uncomfortable; and 4 points: you are very uncomfortable. The results range from 0 to 88 points, and higher scores indicate a worse quality of life [25].
2. **A custom-designed survey questionnaire**—composed of two sections. The first section (demographic data) included questions about sex and age. The second section contained questions regarding SS dysfunctions, including difficulties with chewing, drinking, eating hard and soft foods, smiling, swallowing, speaking, and maintaining a natural facial expression.

The patient self-assessment in the control group was conducted in a dedicated area within the hospital ward, where the patient was guaranteed privacy when answering. The control group survey was conducted in a secluded, quiet area of the orthodontic clinic to eliminate factors that might distract the patient or influence the response.

### Statistical Analysis

Statistical analyses were conducted using IBM SPSS Statistics 30. Descriptive statistics were calculated along with the Shapiro–Wilk test to examine the distribution of variables and verify the assumption of normality. Student’s *t*-test for independent samples was applied to compare two equal-sized groups in terms of continuous variables. The chi-square test for independence was used to compare two groups based on nominal variables. The Mann–Whitney U test was employed to compare two unequal-sized groups regarding continuous variables. Additionally, Pearson’s correlation analysis was performed to examine relationships between continuous variables. A significance level of α = 0.05 was adopted for this study.

## 3. Results

Table 1 presents the demographic data of the surveyed persons.

In the next stage of the analysis, the distribution of continuous variables was examined. Basic descriptive statistics were calculated, and the Shapiro–Wilk test was performed to assess normality.

The results indicated that the Shapiro–Wilk test was statistically significant for all variables in both patient groups, suggesting that their distributions deviated significantly from normality (Table 2). However, the skewness of these distributions did not exceed an absolute value of 2, indicating only slight asymmetry [26]. Therefore, further analyses were conducted using parametric tests, provided that their other assumptions were met.

### 3.1. A Comparison of the Study and Control Groups

Next, this study examined whether orthognathic patients before surgery (the study group) differed from orthodontically treated patients without surgical intervention (the control group) in terms of quality-of-life assessment. For this purpose, an independent-samples *t*-test was performed to compare the study and control groups concerning overall quality of life and its specific dimensions (facial aesthetics, oral function, awareness of dentofacial deformity, and social aspects).

The analysis revealed statistically significant differences between the groups for all orthodontic–orthognathic quality-of-life indicators. Orthognathic patients before surgery had significantly higher scores, indicating a significantly lower overall quality of life and lower scores in the specific dimensions compared to orthodontically treated patients without surgical intervention (Table 3). Furthermore, all observed effects were strong (Cohen’s d ≥ 0.80).

Subsequently, the frequency of specific stomatognathic system (SS) dysfunctions was compared between orthognathic patients before surgery (the study group) and orthodontically treated patients without surgical intervention (the control group). A chi-square test for independence was used to compare the two groups regarding the frequency of individual SS dysfunctions.

The analysis demonstrated statistically significant differences between the groups in the frequency of difficulties with chewing, drinking, eating hard foods, smiling, speaking, and maintaining a neutral facial expression. Orthognathic patients before surgery reported significantly more frequent difficulties in these areas compared to orthodontically treated patients without surgical intervention (Table 4). Additionally, the effects observed for chewing, smiling, and maintaining a neutral facial expression were strong (ϕ ≥ 0.50), the effect for speaking was moderate (0.30 ≤ ϕ < 0.50), and the effects for drinking and eating hard foods were weak (ϕ < 0.30) (Table 4).

To further analyse the findings, the number of reported SS dysfunctions was compared between the study and control groups. An independent-samples *t*-test was performed.

The analysis revealed a statistically significant difference between the groups in the number of reported SS dysfunctions. Orthognathic patients before surgery reported a significantly greater number of dysfunctions compared to orthodontically treated patients without surgical intervention (Table 5). Additionally, the observed effect was strong (Cohen’s d ≥ 0.80).

### 3.2. Comparison of Patients with Retrognathia and Prognathism

The next step of the analysis examined whether patients with retrognathia (Class II) differed from those with prognathism (Class III) in terms of quality-of-life assessment. An independent-samples *t*-test was used to compare these two groups concerning overall quality of life and its specific dimensions (facial aesthetics, oral function, awareness of dentofacial deformity, and social aspects).

The analysis did not reveal statistically significant differences between the two groups in any of the orthodontic–orthognathic quality-of-life indicators. This indicated that patients with retrognathia did not differ from those with prognathism in terms of overall quality of life or specific dimensions related to social aspects of facial deformity, facial aesthetics, oral function, and awareness of facial deformity (Table 6).

Next, the frequency of specific stomatognathic system dysfunctions was compared between patients with prognathism and those with retrognathia. A chi-square test for independence was used to compare the two groups in terms of the frequency of individual SS dysfunctions.

The analysis did not reveal statistically significant differences between the groups in the frequency of specific difficulties with the masticatory system. This indicated that regardless of whether patients had retrognathia or prognathism, they exhibited similar frequencies of difficulties with chewing, drinking, eating hard and soft foods, smiling, swallowing, speaking, and maintaining a neutral facial expression (Table 7).

To expand upon this analysis, the number of reported SS dysfunctions was compared between patients with retrognathia and those with prognathism. An independent-samples *t*-test was performed.

The analysis did not reveal a statistically significant difference between the compared groups in terms of the number of reported stomatognathic system (SS) dysfunctions. This indicated that patients with retrognathia did not differ from those with prognathism regarding the number of declared dysfunctions (Table 8).

### 3.3. Association Between Sex, Age, and Quality of Life Assessment

The final stage of the analysis examined whether sex and age were associated with quality-of-life assessment among orthognathic patients. First, a comparison was made between women and men before orthognathic surgery in terms of overall quality-of-life assessment and its specific dimensions. Due to the statistically significant imbalance between the compared groups (χ^2^(1) = 4.95; *p* = 0.026), an analysis was conducted using the nonparametric Mann–Whitney test.

The analysis revealed a statistically significant difference between the groups in overall quality-of-life assessment, social aspects of facial deformity, and facial aesthetics. Women had significantly higher scores, indicating a significantly lower quality-of-life assessment in overall quality of life, social aspects of facial deformity, and facial aesthetics compared to men. Additionally, the observed effects were moderate (0.06 ≤ η^2^ < 0.14) (Table 9).

Next, the relationship between age and overall and specific dimensions of quality-of-life assessment in orthognathic patients was examined using Pearson’s correlation analysis.

The analysis did not reveal statistically significant associations between age and overall or specific dimensions of quality-of-life assessment (Table 10). This indicated that changes in patient age in the study group did not correlate with changes in their assessment of overall quality of life or specific dimensions, including social aspects of facial deformity, facial aesthetics, oral function, and awareness of facial deformity.

## 4. Discussion

This study assessed two key aspects concerning orthognathic patients. The first focused on quality of life, evaluated using the standardized OQLQ questionnaire, which is a widely recognized tool for assessing the quality of life in patients with maxillofacial deformities [27]. The second aspect involved self-reported stomatognathic system (SS) dysfunctions in these patients. Previous research on this topic has been conducted with healthy control groups; however, we did not find studies in which the control group consisted of orthodontically treated patients without indications for surgical intervention [28]. In our view, comparing these groups in terms of quality of life across four key domains—facial aesthetics, oral function, awareness of dentofacial deformity, and social aspects—is particularly valuable. The findings of this study may help specialists in maxillofacial surgery and orthodontics better understand the impact of skeletal facial deformities on both the somatic and psychological functioning of patients.

The results of this study revealed statistically significant differences between the compared groups across all OQLQ indicators. Orthognathic patients before surgery exhibited significantly lower overall and domain-specific quality of life compared to orthodontically treated patients without surgical intervention. Notably, all observed effects were strong (Cohen’s d ≥ 0.80). These findings suggest that patients with dentoskeletal deformities are more dissatisfied with their health status than patients with malocclusions that do not require surgical correction. Consequently, orthognathic surgery is often necessary not only for aesthetic reasons but also for health and even life-preserving purposes. Similar conclusions have been drawn by other researchers in studies where the control group consisted of healthy individuals [28].

In the next phase of our research, we plan to evaluate the quality of life of orthognathic patients after surgery to assess its impact on their daily functioning.

Another key aspect analysed was the presence of SS dysfunctions reported by patients before surgery and their comparison with orthodontically treated patients without surgical indications. Our study found that orthognathic patients before surgery reported significantly more SS dysfunctions than orthodontically treated patients. Surgical patients more frequently reported difficulties in areas such as food chewing, drinking, eating hard foods, smiling, speaking, and maintaining a neutral facial expression compared to orthodontic patients. Importantly, the effects observed for chewing, smiling, and maintaining a neutral facial expression were strong (ϕ ≥ 0.50), the effect for speaking was moderate (0.30 ≤ ϕ < 0.50), and the effects for drinking and eating hard foods were weak (ϕ < 0.30). A study by Hnitecka et al. demonstrated that over 39% of orthognathic patients reported discomfort related to chewing and swallowing before surgery. Therefore, a thorough preoperative examination of the SS is crucial for identifying functional deficits in orthognathic patients [29].

The study also compared surgical patients with retrognathia (Class II) and prognathism (Class III) in terms of overall and domain-specific quality of life and SS dysfunctions. The findings indicated that patients with retrognathia did not differ from those with prognathism in terms of quality of life or the frequency and number of SS dysfunctions. However, Sen et al. reported different findings, demonstrating that Class III patients had significantly higher OQLQ scores in the domains of oral function, awareness of dentofacial deformity, and social aspects [30]. In clinical practice, it is often observed that patients with prognathism more frequently face negative perceptions of their facial profile compared to those with retrognathia [31].

Scientific data suggest that women are more likely to undergo orthognathic surgery than men [32]. In this study, we examined whether sex and age were associated with quality-of-life assessment among orthognathic patients. The results showed that women had significantly lower overall quality of life scores and poorer assessments of the social aspects of facial deformity and facial aesthetics compared to men (0.06 ≤ η^2^ < 0.14). Similar findings were reported by Stagles et al., who observed a lower preoperative quality of life in women than in men [33]. Additionally, Sen et al. found that women in the surgical group had significantly higher scores in the domains of dentofacial aesthetic awareness and social disability [30]. Our results are also consistent with the findings of Bortoluzzi et al., who concluded that women had poorer quality of life compared to men across all OQLQ domains, particularly in facial aesthetics, oral function, and awareness of facial deformity [34].

Our study did not find statistically significant associations between age and quality-of-life assessment in orthognathic patients. However, Bortoluzzi et al. reported contrasting findings, demonstrating that the negative impact on quality of life increases with age, particularly in the domains of facial aesthetics and oral function [34].

One of the most important determinants of an individual’s behaviour and functioning is their personality. According to the literature, the psychological characteristics of the individual may be crucial in the diagnostic and treatment process of orthognathic patients [35]. Studies show that patients who undergo orthognathic surgery tend to have anxious traits, excessive concern about their bodies and health that is not supported by an organic basis, tend to be insecure and inhibited, and have relationship difficulties. They are extroverted; they tend to focus on the present; and they face life in a planned way [36]. Therefore, an assessment of the patient’s emotional state before and after surgery is essential, as postoperative satisfaction and rehabilitation are supported by psychological well-being [37].

Therefore, referring to the hypothesis and analysing the results obtained, it can be stated with great caution that patients with malocclusion qualified for surgery, due to the complexity of the health problem, present a worse quality of life than patients undergoing conventional orthodontic treatment. In addition, they are characterized by a higher incidence of dysfunction within the stomatognathic system (SS).

There were some limitations to the study, i.e., the small study group, the orthognathic patients were assessed the day before surgery, when the level of perceived stress may have been very high, which therefore may have translated into high scores on the quality of life questionnaire. Another limitation of the study was the bias in patients’ self-assessment.

Long-term longitudinal and multicentre studies are needed for the Polish population. In the future, we would like to conduct a study on a larger group of patients and assess the quality of life after orthognathic surgery both during the recovery period and at 1, 3, 6 and 12 months after surgery.

## 5. Conclusions

Orthognathic patients, especially women, have a lower quality of life and are more likely to have impaired stomatognathic function than patients with malocclusion without indications for surgery, which should be taken into account when qualifying people for surgery.

## Figures and Tables

**Table 1:** The demographic data of patients in the study and control groups.

Dependent Variable	Study Group (n = 73)					Control Group (n = 60)					
	M	Me	SD	Min	Max	M	Me	SD	%	Min	Max
Age	28.56	28	6.37	18	40	25.28	22.50	7.07		18	45
Dependent variable	Gender (%)					Gender (%)					
Male	63					60					
Female	37					40					

Annotation. n—number of observations; M—mean; Me—median; SD—standard deviation; Min—minimum value; Max—maximum value; %—percent.

**Table 2:** Basic descriptive statistics of the study variables with the Shapiro–Wilk test (N = 133).

Variable	M	Me	SD	Sk	Kurt	Min	Max	W	*p*
Study group (n = 73)									
General evaluation of **OQLQ**	40.47	37.00	16.38	0.53	−0.33	12.00	83.00	0.97	**0.050**
Social aspects of facial deformity	11.75	11.00	8.19	0.74	−0.20	0.00	32.00	0.94	**0.001**
Facial aesthetics	13.42	14.00	4.59	−0.47	−0.71	3.00	20.00	0.95	**0.004**
Oral function	8.38	8.00	5.01	0.46	−0.34	0.00	20.00	0.96	**0.038**
Awareness of facial deformity	7.08	6.00	4.34	0.29	−0.66	0.00	16.00	0.96	**0.022**
Number of impairments in stomatognathic function	2.90	3.00	1.67	0.30	−0.63	0.00	6.00	0.93	**<0.001**
Control group (n = 60)									
General evaluation of QofL	11.63	11.00	3.22	0.62	−0.19	5.00	20.00	0.94	**0.005**
Social aspects of facial deformity	2.08	2.00	1.23	0.47	0.91	0.00	6.00	0.92	**<0.001**
Facial aesthetics	3.60	3.00	1.68	0.80	0.63	1.00	8.00	0.91	**<0.001**
Oral function	4.08	4.00	1.09	0.63	−0.59	3.00	7.00	0.84	**<0.001**
Awareness of facial deformity	1.93	1.00	2.20	1.40	1.44	0.00	9.00	0.81	**<0.001**
Number of disorders of the SS	0.62	0.00	0.83	1.39	1.56	0.00	3.00	0.72	**<0.001**

Annotation. M—mean; Me—median; SD—standard deviation; Sk.—skewness; Kurt.—kurtosis; Min—minimum value; Max.—maximum value; W—Shapiro–Wilk test result; *p*—statistical significance of Shapiro–Wilk test.

**Table 3:** A comparison of patients in the study group with patients in the control group in terms of total and detailed quality of life dimensions (N = 133).

	Study Group (n = 73)		Control Group (n = 60)					95% CI		
Dependent Variable	M	SD	M	SD	t	df	*p*	LL	UL	d Cohena
General evaluation of **OQLQ**	40.47	16.38	11.63	3.22	14.70 ^a^	78.70	**<0.001**	24.93	32.74	2.34
Social aspects of facial deformity	11.75	8.19	2.08	1.23	9.96 ^a^	75.91	**<0.001**	7.74	11.60	1.58
Facial aesthetics	13.42	4.59	3.60	1.68	16.95 ^a^	94.30	**<0.001**	8.67	10.98	2.74
Oral function	8.38	5.01	4.08	1.09	7.13 ^a^	80.27	**<0.001**	3.10	5.50	1.14
Awareness of facial deformity	7.08	4.34	1.93	2.20	8.85 ^a^	110.87	**<0.001**	4.00	6.30	1.45

Annotation. n—number of observations; M—mean; SD—standard deviation; t—value of test statistic; df—degrees of freedom; *p*—statistical significance; CI—confidence interval for the difference between means; LL and UL—lower and upper limits of the confidence interval. ^a^ Levene’s test result was found to be statistically significant—the result with Welch’s correction was reported.

**Table 4:** A comparison of patients in the study group with patients in the control group in terms of the frequency of individual impairments of stomatognathic function (N = 133).

In Which Activities Does Your Current Chewing Organ Problem Hinder You?		Study Group		Control Group				
		N	%	N	%	χ^2^(1)	*p*	ϕ
Chewing	No	30	41.1%	57	95.0%	42.30	**<0.001**	0.56
	Yes	43	58.9%	3	5.0%			
Drinking	No	67	91.8%	60	100.0%	5.16	**0.032**	0.20
	Yes	6	8.2%	0	0.0%			
Eating hard foods	No	31	42.5%	40	66.7%	7.75	**0.009**	0.24
	Yes	42	57.5%	20	33.3%			
Eating soft foods	No	70	95.9%	58	96.7%	0.06	1.000	0.02
	Yes	3	4.1%	2	3.3%			
Smiling	No	28	38.4%	57	95.0%	45.81	**<0.001**	0.59
	Yes	45	61.6%	3	5.0%			
Swallowing	No	70	95.9%	60	100.0%	2.52	0.252	0.14
	Yes	3	4.1%	0	0.0%			
Speaking	No	49	67.1%	56	93.3%	13.61	**<0.001**	0.32
	Yes	24	32.9%	4	6.7%			
Maintaining a normal facial expression	No	27	37.0%	55	91.7%	41.65	**<0.001**	0.56
	Yes	46	63.0%	5	8.3%			

Annotation. N—number of observations; χ^2^—chi-square test result; *p*—statistical significance; ϕ—effect strength index.

**Table 5:** A comparison of patients in the study group with patients in the control group in terms of the number of impairments of stomatognathic function (N = 133).

	Study Group (n = 73)		Control Group (n = 60)					95% CI		
Dependent Variable	M	SD	M	SD	t	df	*p*	LL	UL	d Cohena
Number of disorders of stomatognathic system	2.90	1.67	0.62	0.83	10.28 ^a^	109.43	**<0.001**	1.85	2.73	1.69

Annotation. n—number of observations; M—mean; SD—standard deviation; t—value of test statistic; df—degrees of freedom; *p*—statistical significance; CI—confidence interval for the difference between means; LL and UL—lower and upper limits of the confidence interval. ^a^ Levene’s test result was found to be statistically significant—the result with Welch’s correction was reported.

**Table 6:** Comparison of retrogenic patients with progenic patients in terms of quality of life overall and in specific dimensions (N = 73).

	Retrogenia (n = 34)		Progenia (n = 39)					95% CI		
Dependent Variable	M	SD	M	SD	t	df	*p*	LL	UL	d Cohena
General evaluation of **OQLQ**	40.53	17.24	40.41	15.81	0.03	71	0.976	−7.60	7.84	<0.01
Social aspects of facial deformity	11.76	8.38	11.74	8.12	0.01	71	0.991	−3.84	3.88	<0.01
Facial aesthetics	13.29	4.69	13.54	4.56	−0.23	71	0.822	−2.41	1.92	0.05
Oral function	9.00	5.37	7.85	4.68	0.98	71	0.330	−1.19	3.50	0.23
Awareness of facial deformity	6.91	4.72	7.23	4.03	−0.31	71	0.756	−2.36	1.72	0.07

Annotation. N—number of observations; M—mean; SD—standard deviation; t—value of test statistic; df—degrees of freedom; *p*—statistical significance; CI—confidence interval for the difference between means; LL and UL—lower and upper limits of the confidence interval. Levene’s test result was found to be statistically significant—the result with Welch’s correction was reported.

**Table 7:** Comparison of patients with retrogenia with patients with progenia in terms of the frequency of individual impairments of stomatognathic function (N = 73).

In Which Activities Does Your Current Chewing Organ Problem Hinder You?		Retrogenia		Progenia				
		N	%	N	%	χ^2^(1)	*p*	ϕ
Chewing	No	12	35.3%	18	46.2%	0.89	0.475	0.11
	Yes	22	64.7%	21	53.8%			
Drinking	No	31	91.2%	36	92.3%	0.03	1.000	0.02
	Yes	3	8.8%	3	7.7%			
Eating hard foods	No	14	41.2%	17	43.6%	0.04	1.000	0.02
	Yes	20	58.8%	22	56.4%			
Eating soft foods	No	32	94.1%	38	97.4%	0.51	0.595	0.08
	Yes	2	5.9%	1	2.6%			
Smiling	No	11	32.4%	17	43.6%	0.97	0.347	0.12
	Yes	23	67.6%	22	56.4%			
Swallowing	No	33	97.1%	37	94.9%	0.22	1.000	0.05
	Yes	1	2.9%	2	5.1%			
Speaking	No	19	55.9%	30	76.9%	3.64	0.081	0.22
	Yes	15	44.1%	9	23.1%			
Maintaining a normal facial expression	No	16	47.1%	11	28.2%	2.77	0.144	0.19
	Yes	18	52.9%	28	71.8%			

Annotation. N—number of observations; χ^2^—chi-square test result; *p*—statistical significance; ϕ—effect strength index.

**Table 8:** A comparison of patients with retrogenia with patients with progenia in terms of the number of impairments of stomatognathic function (N = 73).

	Retrogenia (n = 34)		Progenia (n = 39)					95% CI		
Dependent Variable	M	SD	M	SD	t	df	*p*	LL	UL	d Cohena
Number of disorders of stomatognathic system	3.06	1.84	2.77	1.51	0.74	71	0.463	−0.49	1.07	0.17

Annotation. n—number of observations; M—mean; SD—standard deviation; t—value of test statistic; df—degrees of freedom; *p*—statistical significance; CI—confidence interval for the difference between means; LL and UL—lower and upper limits of the confidence interval. Levene’s test result was found to be statistically significant—the result with Welch’s correction was reported.

**Table 9:** A comparison between men and women in the study group in terms of their assessment of quality of life overall and in specific dimensions (N = 73).

	Women (n = 46)			Men (n = 27)					
Dependent Variable	Mean Rank	M	SD	Mean Rank	M	SD	Z	*p*	η^2^
General evaluation of **OQLQ**	40.89	43.74	17.46	30.37	34.89	12.81	−2.05	**0.041**	0.06
Social aspects of facial deformity	41.12	13.28	8.58	29.98	9.15	6.87	−2.17	**0.030**	0.07
Facial aesthetics	40.78	14.26	4.37	30.56	12.00	4.68	−2.00	**0.046**	0.06
Oral function	39.45	9.00	5.52	32.83	7.33	3.86	−1.29	0.198	0.02
Awareness of facial deformity	38.00	7.37	4.63	35.30	6.59	3.83	−0.53	0.598	<0.01

Annotation. n—number of observations; M—mean; SD—standard deviation; Z—value of test statistic; *p*—statistical significance; η^2^—strength of effect index.

**Table 10:** Correlation of age with assessment of quality of life overall and in specific dimensions (N = 73).

	Age	
Variable	r Pearsona	*p*
General evaluation of **OQLQ**	0.01	0.932
Social aspects of facial deformity	−0.06	0.601
Facial aesthetics	0.10	0.391
Oral function	−0.05	0.656
Awareness of facial deformity	0.10	0.377

## Data Availability

The data presented in this study are available upon request from the corresponding author. The data are not publicly available due to sensitive information.
